# Predicting Coping With Self-Regulated Distance Learning in Times of COVID-19: Evidence From a Longitudinal Study

**DOI:** 10.3389/fpsyg.2021.701255

**Published:** 2021-09-03

**Authors:** Fred Berger, Claudia Schreiner, Wolfgang Hagleitner, Livia Jesacher-Rößler, Susanne Roßnagl, Christian Kraler

**Affiliations:** ^1^Institute of Education, University of Innsbruck, Innsbruck, Austria; ^2^Department for Teacher Education and School Research, University of Innsbruck, Innsbruck, Austria

**Keywords:** COVID-19, coping with self-regulated learning, distance learning, educational inequality, academic achievement, learning motivation, support at home and from school, longitudinal study

## Abstract

Due to the COVID-19 pandemic, students worldwide have experienced fundamental changes to their learning. Schools had to shift to distance education as part of the effort to stop the spread of the virus. Although distance learning undoubtedly resulted in challenges for all students, there is much concern that it exacerbated existing educational inequalities and led to disadvantages – particularly for students who were already struggling academically and lacking support from family and school. The aim of this paper was to investigate the possible impact of family and child characteristics, school performance prior to lockdown, and support at home and from school during lockdown in coping with self-regulated distance learning during times of COVID-19. The paper draws on data from a two-wave longitudinal study surveying 155 lower secondary school students aged 13–14years from a rural-alpine region in Austria. Data were collected 1year before the start of the pandemic and directly after schools had returned to in-class teaching after the first lockdown. Our findings support the notion that distance learning poses a substantial risk for exacerbating existing educational disadvantages. They show that coping with out-of-school learning was especially challenging for students with low academic achievement and learning motivation prior to the pandemic. Furthermore, findings demonstrate that the support from parents and teachers foster students’ capabilities to cope with the self-regulatory demands connected with distance learning. Although the importance of competencies for self-regulated learning became particularly evident in the context of the pandemic, from our findings, it can be concluded that in the future, schools should strengthen their investment in promoting competencies for self-regulated learning. Self-regulation must be recognized as an essential educational skill for academic achievement and life-long learning.

## Introduction

Shortly after the World Health Organization (2020) had declared the Coronavirus disease outbreak a pandemic on March 11, 2020, many countries around the world instigated temporary closures of schools and universities as part of an effort to prevent and slow down transmission of the virus. By the end of April 2020, more than 1.5 billion students (over 90% globally) had been affected by the closure of schools and higher education institutions in response to the COVID-19 pandemic (UNESCO, 2021). As a consequence of these shutdowns, students were confronted with abrupt changes, both in their learning and their daily lives as a whole. Distance learning requires a large amount of self-regulation, potentially putting students at risk of missing out on wider learning opportunities and of being overwhelmed by the requirements to acquire and understand academic content with reduced or minimal support from their teachers (Pelikan et al., 2021). Moreover, the lack of physical presence and the lesser extent of informal discourse and spontaneous interaction with classmates, friends, and teachers increase the risk of developing negative emotions and feelings of loneliness.

In this study, we seek to examine how students managed to cope with the new challenges of self-regulated distance learning during school closures. Although involuntary distance learning created challenges for all students, based upon previous studies and theoretical considerations of the development of educational inequalities (Maaz et al., 2010; APA, 2020; Grewenig et al., 2020; Huber and Helm, 2020; SRCD, 2020), we argue that school closures did not affect all students to the same extent. We assert that distance learning exacerbated existing vulnerabilities and led to increased disadvantages in learning for particular groups of students. In particular, we assume increased disadvantages for those students with low levels of academic achievement and learning motivation and with low competence in self-regulation prior to lockdown and for those students with little support at home or from school during the shutdown.

Our analyses draw upon data from a two-wave longitudinal study with students from an alpine region of Austria. Students were in seventh grade during wave 1 and in eighth grade during wave 2 (aged approx. 13–14years). Data were collected in May 2019, 1year before the start of the pandemic, and in June and July 2020, right after Austrian schools had reopened after the first lockdown from March 16 to May 18, 2020.

Since the equipment for and use of digital media in Austrian schools was rather low in comparison to schools of other European countries (OECD, 2020), the involuntary shift to distance learning in March 2020 was especially challenging. In many cases, models and strategies for distance learning had to be developed first (Jesacher-Rößler and Klein, 2020). Furthermore, due to the regulations during the first strict lockdown, schools were only allowed to provide in-school education for students whose parents were key workers (BMBWF, 2020). Therefore, schools could not ask specific groups of students, for example, low achieving and low motivated students or students with little support at home, to come into school in order to support them.

### Theoretical Considerations on the Link Between Self-Regulated Learning and Academic Achievement

In this article, the term self-regulated learning refers to a learner’s competence to plan, execute, and evaluate his or her learning autonomously (Wirth and Leutner, 2008). Self-regulation is seen as a dynamic and cyclical process, which involves the active interpretation of tasks, goal setting, making plans, identifying strategies that will ensure success, and constantly monitoring and readjusting one’s learning toward the attainment of set goals (Schunk and Greene, 2018). In the research literature, self-regulation is recognized as a developmental process that begins well before children enter formal schooling. It must be learned over the course of life and is considered to be an essential educational skill, especially with regard to academic achievement and life-long learning (Illeris, 2009; Usher and Schunk, 2018). There is a substantial body of literature, which links self-regulation to academic achievement; identifying it as a major cause and consequence of achievement gaps among students across educational levels and settings, even after controlling for previous achievement, IQ, and demographic characteristics (Cleary and Zimmerman, 2006; Zimmerman and Schunk, 2011; Jacob and Parkinson, 2015; Edossa et al., 2017; Perry et al., 2018). Students who struggle with self-regulation often show low academic achievement and vice versa. Furthermore, they frequently display low self-esteem and demonstrate low self-efficacy for changing outcomes in their lives (Cleary and Zimmerman, 2006; Zimmerman and Schunk, 2011; Perry et al., 2018).

Although there are several theoretical perspectives on self-regulated learning, these perspectives share common features (Pandero, 2017). One central feature is the key role of *motivation*. It is considered to be critical in both directing self-regulation and in maintaining energy to achieve goals (Schunk and Zimmerman, 2008; Efklides, 2011). A learner’s motivation predicts his/her willingness to engage in cycles of strategic actions to facilitate learning (Zimmerman, 2008). Without the motivation to act and to “sustain the behaviors necessary, potentiality remains just that” (Usher and Schunk, 2018, p. 27).

A second common feature is the strong influence of *contextual factors*. Supportive contexts and relationships can help learners to withstand challenges that might otherwise overwhelm them and can assist them in developing self-regulation competency. In particular, support from parents and teachers is considered as very important in fostering self-regulated learning. Home and school environments, where children and adolescents experience authoritative forms of parenting and teaching (e.g., warmth and responsiveness, support of autonomy, and scaffolding), are likely to exert a positive influence on learners’ own development of self-regulation (Eisenberg et al., 2009; Perry et al., 2018). In contrast, where low levels of instructional support and organizational qualities exist at home or at school, this can negatively impact children’s development of self-regulation (Perry et al., 2018). Children gradually assume control of their thoughts and actions when they grow up in emotionally safe surroundings, which support their autonomy and by learning from parents and teachers as positive role models for self-regulation (Schunk and Zimmerman, 1997; Rimm-Kaufman et al., 2009). Specifically in classrooms, instructional emphases on higher-order thinking, talking about learning and engaging students in meaningful work with formative feedback have been shown to support students’ development of self-regulation (Perry, 2013). Self-regulated learning is very much shaped through the personal agency of the teacher who introduces and reinforces self-regulated learning experiences and offers scaffolded instructions and feedback, which help students to master their tasks and to meet their goals (Bembenutty, 2013).

### Previous Findings on Students’ Coping With Self-Regulated Learning in Times of COVID-19

There is a fast-growing body of research on the topic of students’ ability to cope with distance learning in times of COVID-19 (e.g., see the systematic review of Helm et al., 2021). However, these results need further confirmation. To date, they show a wide range of variation. One emerging finding from research conducted so far is that most students coped quite well with the challenges of distance learning in 2020. Analyses of out-of-school learning in German-speaking countries (Germany, Austria, and Switzerland) indicate that 37–70% of students, depending upon study design, age, and school level, liked remote learning at home (Huber et al., 2020: 37%; Trültzsch-Wijnen and Trültzsch-Wijnen, 2020: 46%; Holtgrewe et al., 2020: 55%; Baier and Kamenowski, 2020: 55%; Schwerzmann and Frenzel, 2020: 70%). Moreover, 42% (special school) to 70% (grammar school) of students report having been more self-regulated in times of distance learning than in times of face-to-face teaching (Schwerzmann and Frenzel, 2020).

However, findings also indicate that a substantial number of students had difficulties coping with the different requirements of remote learning. Twenty-two to forty-four percent of the students described that they had to cope with problems during lockdown: Many students declared that they had difficulties organizing their self-regulated learning (Letzel et al., 2020: 35%; Huber and Helm, 2020: 44%), felt overwhelmed by the requirements of distance learning (Holtgrewe et al., 2020: 35%), reported having problems with concentrating on their tasks (Schwerzmann and Frenzel, 2020: 36%) or described that it was difficult for them to do their homework on their own (Holtgrewe et al., 2020: 22%; ADAS and LIFE, 2020: 26%). With regard to school performance, a substantial proportion of students expected distance learning to have negative effects on their academic achievement (Schwerzmann and Frenzel, 2020: 20%; Baier and Kamenowski, 2020: 38%; Refle et al., 2020: 40%; Anger et al., 2020: 45%; Trültzsch-Wijnen and Trültzsch-Wijnen, 2020: 50%). Furthermore, a significant percentage of students from different school phases reported low levels of home learning (a maximum of 2h a day) during the lockdown (Huber et al., 2020: 24%; Anger et al., 2020: 35%; Wacker et al., 2020: 45%).

Additional research indicated that most students aged 10–19 felt well-supported at home and from school during lockdown (Helm et al., 2021). According to an Austrian study, only 3% of students reported that they did not know how to contact their teachers when they had questions about their distance learning (Schober et al., 2020). Fifty-three percent reported having weekly contact, and 41% having daily contact with their teachers (Trültzsch-Wijnen and Trültzsch-Wijnen, 2020). Nonetheless, a substantial proportion of students missed out on having any contact or support with school-related tasks from their teachers (Letzel et al., 2020: 22%; Holtgrewe et al., 2020: 38%; Wacker et al., 2020: 43%; Baier and Kamenowski, 2020: 42.6–49%). At home, most support (about 60–80%) was provided by mothers (Heller and Zügel, 2020: 84%; Schober et al., 2020: 59%; Wildemann and Hosenfeld, 2020: 81%). One-fifth to one-third of the students reported a lack of support when learning at home (Schober et al., 2020: 21%; Refle et al., 2020: 33%), but only 10% would have appreciated more support from their parents (Letzel et al., 2020).

### Previous Findings on Differences in Coping With Self-Regulated Distance Learning

Many scholars have argued that children from families with few resources and support at home and with low academic achievement and learning motivation might be disadvantaged by distance learning in times of COVID-19 (e.g., Grewenig et al., 2020; Huber and Helm, 2020; SRCD, 2020). Out-of-school learning may thus increase educational inequalities and widen the educational gap between children from different family backgrounds and with different school performance prior to the pandemic. Although there are only limited findings available on this topic, existing results lend support to this notion.

Regarding family characteristics, research findings suggest that students from disadvantaged backgrounds experienced more barriers to learning success than students from privileged families (Anger et al., 2020; Baier and Kamenowski, 2020; Steiner et al., 2020; Thies and Klein, 2020; Vuorikari et al., 2020; Wößmann et al., 2021). During home-schooling, socioeconomic status and available resources of the family gained in importance with respect to learning achievement (Berghammer, 2020). In their systematic review, Helm et al. (2021) concluded that there are positive correlations between socioeconomic background and learning success, motivation, technical equipment at home, and parental support during distance learning. Furthermore, studies conducted in the United States indicated that cultural differences and limited linguistic proficiency of students and parents in the national language reduced participation in distance learning during school shutdowns (SRCD, 2020).

However, in the context of distance learning, it could also be shown that not only domestic factors but also school-related factors play a role in connection with disadvantages. The quality of teacher-student interaction and a teacher’s competence in applying appropriate distance learning pedagogy as well as to provide timely and informative feedback turned out to be predictive for differences in learning success and learning investment of students during school shutdown (Huber and Helm, 2020).

With respect to gender differences, female students were found to show higher learning engagement and to receive more support from teachers than male students (Korlat et al., 2021). This finding correlates with results from previous studies, which indicate that, in general, girls are more proficient at self-regulated learning than boys (Perry et al., 2018).

Finally, in their study on how school closure affected low- and high-achieving students, Grewenig et al. (2020) demonstrate that while self-regulated learning is feasible for high achieving and high-motivated students, it is especially challenging for students with low academic achievement and learning motivation. The latter often lack the knowledge and skills as well as the energy and persistence in task-oriented behavior necessary to generate additional learning gains through self-regulated distance learning. While students on average reduced their daily learning time during the school shutdown, low-achievers disproportionately lowered their time on task and replaced it with detrimental activities such as TV or computer games (Grewenig et al., 2020).

### Research Hypotheses

These initial results suggest that school shutdowns might have exacerbated existing vulnerabilities and led to increased inequalities in education, particularly for those students already at risk of low levels of school achievement and learning motivation. They make a strong case for further focus on students’ ability to cope with self-regulation in times of distance learning, specifically examining the possible impact of family background, support from parents and schools, and academic achievement and learning motivation on exacerbating existing educational inequalities. In this study, we will therefore take a closer look at these potential determinants of students’ ability to cope with distance learning during school shutdown. Based upon the above findings and theoretical considerations concerning self-regulation and the development of educational inequalities, we propose that academic achievement and learning motivation prior to school shutdown may predict ability to cope with self-regulated learning during distance learning. Furthermore, we anticipate emotional support from parents and accessibility of teachers during lockdown to foster students’ capabilities to cope with the self-regulatory demands connected with distance learning. Parental education, students’ first language, and gender are included in the analyses to take into account family and child characteristics. We hypothesize that students from more highly educated parents, students with German as their first language, and girls will have an advantage in coping with the challenges of out-of-school self-regulatory learning.

In the results section, we will first present descriptive statistics in order to characterize the students’ situation during spring 2020s lockdown and distance learning due to the first wave of COVID-19 in Austria (section “Students’ Perceptions of Lockdown, Distance Learning and Coping With Related Demands”). This includes information on school-related efforts to support distance learning; the core concept of students’ self-regulatory strategies in coping with the demands of distance learning; students’ perceptions of loneliness and social isolation during lockdown and factors of general well-being. Additionally, bivariate correlations show how selected variables describing the students’ circumstances during lockdown are connected to coping with self-regulated distance learning. In section “Effects of Learning Motivation and Academic Achievement on Coping With Distance Learning,” differences between mean-scores in coping with self-regulated distance learning by learning motivation as well as academic achievement prior to the lockdown will be analyzed. Finally, in order to test the above-introduced hypotheses, in section “Effects of Family and Child Characteristics, School Performance, Support at Home and From School on Coping With Distance Learning,” we will estimate the effects of different sets of factors on coping with self-regulated distance learning during the school shutdown.

## Materials and Methods

### Study Design and Research Context

Our analyses are based on data from the first two waves of an Austrian longitudinal study. Overall, the study was designed as a longitudinal project to run for 4years comprising four points of measurement in total. The project aims to survey adolescent students’ transition from lower to upper secondary education. First data collection (T1) took place in May 2019, comprising students in seventh grade from the region of Zillertal in the Province of Tyrol, a rural-alpine region in Austria. The second wave (T2) in eighth grade had originally been scheduled for spring 2020. However, shortly before the data collection was to take place, schools, teachers, and students were faced with the requirement to shift to distance learning mode for several weeks due to the COVID-19 pandemic. Therefore, data collection had to be postponed to June and July 2020 after schools had reopened in Austria. All data were collected using paper-based questionnaires.

The next waves of the longitudinal study (T3 and T4) are planned for the summer terms 2021 and 2022, respectively when students will be in ninth and 10th grades and will have transferred to upper secondary schools.

### Sample

In 2019 and 2020, all seven lower secondary schools in the region took part in the study, yielding a response rate of 100% at school level. Approximately 75% of all seventh grade students (*n*=231) and approx. 75% of the eighth grade students (*n*=234) participated in the study. One hundred and fifty-five students took part in both waves, providing the database for our analyses. Response rate for participation in both waves amounted to 50%. Table 1 comprises the demographic characteristics of the achieved sample.

**Table 1:** Demographic characteristics of the sample.

Gender, *n* (%)	
Female	79 (51.0)
Male	76 (49.0)
Age, *M* (SD)	
T1 (2019)	13.3 (0.42)
T2 (2020)	14.4 (0.41)
First language, *n* (%)	
German (national language)	148 (95.5)
Other languages	7 (4.5)
Parental education^a^, *n* (%)	
Compulsory education only	16 (10.3)
Vocational training	88 (56.8)
School leaving exam K12/13	35 (22.6)
University degree	16 (10.3)

a Highest level of qualification of both parents.

The sample is representative for the population of students in the region being studied (data source: IQS, 2019). However, the population of adults in this region, and thus of parents of the surveyed students, is characterized by a lower level of education, a lower proportion of immigrants and people not speaking the national language (German) as their first language compared to the whole Province of Tyrol as well as to Austrian national levels with, e.g., only 5.6% of the population aged between 25 and 64 in the region having a university degree compared to 14.1% in the Province of Tyrol and 15.8% nationally (Statistics Austria, 2021). Thus, the sample reflects the specific nature of the sub-region as a model for a rural-alpine area with deep, remote and tourist valleys.

Participants reported on their family situation and parent-child relationships, school experiences and academic performance, as well as on their preparation for the transition to upper secondary education and coping with distance learning during the lockdown. All information collected was self-reported by students.

During the lockdown, participants in our study were nearing completion of their lower secondary education (at the end of grade 8) and would very soon be facing the transition to upper secondary education. In addition to the challenges caused by distance learning, they were also confronted with the demanding situation of having to deal with the uncertainty and potential emotional stress connected with the transition process.

### Measures

#### Coping With Self-Regulated Distance Learning in Times of COVID-19

The scale “Coping with self-regulated distance learning in times of COVID-19,” constructed by the authors, was collected in the 2020 survey using four Likert-type four-level items (4=“often,” 3=“sometimes,” 2=“rarely,” 1=“never”; see Table 2B), by using a mean score across the four items. Items 1 and 4 were recoded. Cronbach’s *α* for the scale was 0.79.

**Table 2:** Descriptive statistics on students’ perceptions of distance learning and lockdown.

	Valid *n*	*M*	SD	% Often	% Sometimes	% Seldom		% Never
A. School-related efforts to support distance learning								
(A1) I could easily get in touch with my teachers	155	1.63	0.83	55.5	29.7	11.0		3.9
(A2) Getting access to necessary learning materials was easy	154	1.86	1.03	50.0	25.3	13.6		11.0
(A3) I struggled with digital media	155	2.71	1.05	15.5	27.1	28.4		29.0
B. Coping with self-regulated distance learning								
(B1) It was difficult for me to structure my everyday learning	155	2.21	1.12	17.4	22.6	23.9		36.1
(B2) Learning was easy for me	155	3.06	0.96	41.9	29.7	21.3		7.1
(B3) I could master the tasks assigned to me without problems	154	3.34	0.74	49.4	36.4	13.6		0.6
(B4) Learning at home was difficult for me	155	2.06	1.03	11.6	21.3	29.0		38.1
Scale: Coping with self-regulated distance learning (B1 and B4 recoded)	155	3.03	0.77					
C. Indicators of loneliness and social isolation during lockdown and home schooling								
(C1) I was bored	155	2.43	1.05	22.6	31.6	25.8		20.0
(C2) I felt lonely	155	3.26	0.81	2.6	14.8	36.1		46.5
(C3) I could maintain contact with my friends well	154	1.47	0.74	64.3	27.3	5.2		3.2
(C4) I was longing to get back to school	155	2.17	1.01	29.7	37.4	18.7		14.2
D. Well-being during distance learning	Valid *n*	*M*	SD	% very well (5)	% (4)	% (3)	% (2)	% very unwell (1)
(D1) Some people feel comfortable in their own skin, and others feel less comfortable. How about you?	155	4.19	0.87	43.9	36.1	16.1	3.2	0.6

#### Parent’s Highest Level of Education

The highest completed education of parents was recorded along the ISCED classification and summarized in four categories (Table 1). In two-parent families, the value of the higher educated parent was used. Where necessary, missing values in the 2020 survey year were supplemented by values from the 2019 survey.

#### First Language and Gender

Information on first language and gender of the students was collected from students at both points of measurement.

#### Academic Achievement Prior to Pandemic

Academic achievement is measured on the 5-level grading scale, which is officially used in the Austrian school system (RIS, 2020). To take account of the fact that two different ability groups of students were included in the survey, the scale level is shifted by a value of 2 for students who are assessed in the lower ability assessment group (basic general knowledge – “grundlegende Allgemeinbildung”) compared to students from the higher ability assessment group (in-depth general knowledge – “vertiefte Allgemeinbildung”; Neuweg, 2014). The scale is constructed by calculating the mean of the grades for the subjects German (language of instruction), English (as the first foreign language), and mathematics taken from the winter term certificate, which was issued by the schools in February 2020. This resulted in a seven-point scale, which was transformed so that higher values represented higher achievement (from 1=low performance to 7=high performance; *n*=155; min=1, max=7; M=4.7; SD=1.38; SE=0.11). For group comparisons, students were assigned to one of three groups according to their academic achievement: low (grades up to 3.5; *M*=2.69, SD=0.59), medium (grades higher than 3.5 and up to 5.5; *M*=4.62, SD=0.55), and high academic achievement (grades above 5.5; *M*=6.30, SD=0.49).

#### Learning Motivation Prior to Pandemic

The scale “Learning motivation” (Fend and Prester, 1986) was collected in the 2019 survey and measures learning and achievement-related attitudes using three Likert-type 5-level items (1=“not at all,” 2=“not much,” 3=“moderately,” 4=“fairly,” 5=“very much”; example item: “How persistent are you in completing school tasks?”). The scale score was calculated as a mean across the three items. Cronbach’s *α* for the scale was 0.76 (*n*=155; min/max=1.33/5.00; *M*=3.48; SD=0.80; SE=0.06). For group comparisons, learning motivation was divided into three categories by dividing the students into three groups of approx. equal size: low learning motivation (*M*=1.61, SD=0.22), medium learning motivation (*M*=2.18, SD=0.16), and high learning motivation (*M*=3.10, SD=0.43).

#### Perceived Support and Understanding by Parents During Distance Learning

The scale “Perceived support and understanding by parents” consists of eight items – four items in respect of the mother and four items in respect of the father of the student. The scale measures mothers’ and fathers’ responsiveness to their adolescent child, their understanding and sensitivity to the child’s concerns and problems and their willingness to provide support. The items are derived from the LifE study (Berger and Fend, 2005) and collected with the help of Likert-type 5-level items for father and mother, respectively (1=“do not agree at all,” 2=“agree a little,” 3=“partly/partly,” 4=“agree quite a bit,” 5=“agree completely”; example item: “I feel that I can talk to my father/my mother about anything.”). The scale score was calculated as a mean score from all items for two-parent families and as a mean score from four items for single-parent families (Cronbach’s *α*=0.91; *n*=155; min/max=1.50/5.00; *M*=4.19; SD=0.80; SE=0.06).

#### Teacher Accessibility

The item “I could easily get in touch with my teachers” (1=“never,” 2=“seldom,” 3=“sometimes,” 4=“often”; *n*=155, min/max=1/4, *M*=3.37, SD=0.83, SE=0.07) was constructed by the authors as an indicator of the support that students received from their teachers during distance learning in spring 2020 (see Table 2A1).

#### Single-Item Measures on the Perception of Distance Learning and Lockdown

In addition some single-item measures are used in order to characterize students’ perception of their situations during distance learning and lockdown. Well-being during lockdown was measured on a five-point scale (from “very unwell” to “very well” using emoticons in the form of smileys). School-related efforts to provide help with distance learning were measured with two items on four-point likert scales (4=“often,” 3=“sometimes,” 2=“seldom,” 1=“never”). Possible loneliness and social isolation were assessed by four items on four-point likert scales. The wording and descriptive statistics of all these items are shown in Tables 2A2,A3,C.

### Procedures of Analyses

In order to provide some background information on how the students perceived their situation during distance learning and the lockdown and to test the proposed hypotheses, we applied different methods of statistical analyses. *T*-tests and Mann–Whitney *U*-tests were used to test for gender differences. Spearman’s rho was applied to examine bivariate correlations (section “Students’ Perceptions of Lockdown, Distance Learning, and Coping with Related Demands”).

To test for differences between mean-scores in coping with self-regulated distance learning by learning motivation as well as academic achievement prior to the lockdown, we computed one-way ANOVA in combination with Tukey *post-hoc* tests or Welch-test and Games-Howell *post-hoc* tests. Levene’s tests were used to test for homogeneity of variances (section “Effects of Learning Motivation and Academic Achievement on Coping With Distance Learning”).

In addition, regression analyses were estimated to test the hypotheses formulated (section “Effects of Family and Child Characteristics, School Performance, and Support at Home and From School on Coping with Distance Learning”). To take account of the fact that some variables in the regression models are not normally distributed, analyses were conducted using the bootstrap method in Mplus (Muthen and Muthen, 2017) to estimate bias-corrected standard errors.

## Results

### Students’ Perceptions of Lockdown, Distance Learning, and Coping With Related Demands

#### School-Related Efforts to Facilitate Distance Learning

In spring 2020, teachers and students had to switch to remote teaching and distance learning very quickly and with hardly any time to prepare themselves. Nevertheless, the majority of students in our study (approx. 85%) reported that they could easily contact their teachers for most of the time during lockdown. Access to necessary learning materials was considered to be easy as well. The largest obstacle seemed to be handling digital media, where over 40% of the students reported frequent struggles (Table 2A).

Girls (Mdn=4.0; IQR=3.0–4.0) perceived that it was easier to contact their teachers than boys (Mdn=3.0; IQR=2.2–4.0) (*N*=155; *U*=2184.50; *p*<0.001; Cohen’s *d*=0.54) and that they (Mdn=4.0; IQR=3.0–4.0) had easier access to materials than boys (Mdn=3.0; IQR=2.0–4.0) (*N*=154; *U*=2360.00; *p*<0.05; Cohen’s *d*=0.39). With regard to problems using digital media, there were no gender differences (*N*=155; *U*=2664.99; *p*>0.05).

#### Coping With Self-Regulated Distance Learning

While schools tried to support students learning at home in different ways, the distance-learning situation nevertheless placed specific demands on students. Table 2B shows how the students coped with different self-regulatory demands, which they faced during lockdown and the period of distance learning. In general, most students managed quite well to structure their everyday learning and master the tasks given to them by their teachers.

On average, girls coped better with the self-regulatory demands of distance learning than their male peers. Using the combined scale score on coping with self-regulated distance learning in times of COVID-19, girls showed higher average in coping with self-regulated distance learning (*N*=79; *M*=3.23; SD=0.72) than boys [*N*=76; *M*=2.83; SD=0.76; *t*(153)=−3.366; *p*<0.001; Cohen’s *d*=0.54; variance homogeneity was asserted using the Levene’s test (*p*>0.05)].

#### Loneliness and Social Isolation During Lockdown

While more than half of the students reported feeling bored during lockdown while distance learning, very few reported feeling frequently lonely or having problems keeping in touch with their friends (Table 2C). Nevertheless, the vast majority of students were longing to get back to school.

While girls and boys generally seemed to cope with the social demands of lockdown equally well (being bored: *N*=155; *U*=2853.50; *p*>0.05; maintaining contact with friends: *N*=154; *U*=2609.00; *p*>0.05; feeling lonely: *N*=155; *U*=2549.5; *p*>0.05), girls (Mdn=3.0; IQR=3.0–4.0) reported more often than boys (Mdn=3.0; IQR=2.0–3.0) that they wished they could go back to school (*N*=155; *U*=2085.00; *p*<0.01; Cohen’s *d*=0.57).

Regarding overall well-being, students were asked how they had felt all in all during distance learning and lockdown (Table 2D). The vast majority reported a high level of well-being during this time (43.9% choosing the highest category (“very well”) and another 36.1% the second highest on a five-point scale). Only 0.6% of the students reported feeling very unwell (and another 3.2% choosing the second lowest category). There were no gender differences with regard to self-reported well-being (*N*=155; *U*=3337.00; *p*>0.05).

How well students judged coping with self-regulated distance learning during home schooling most strongly correlated with their general well-being during this period of time (rho=0.520; *p*<0.001). In addition, a high level of ability to cope with self-regulatory demands corresponded with a comparatively low perception of feeling lonely (rho=−0.277; *p*<0.01).

### Effects of Learning Motivation and Academic Achievement on Coping With Distance Learning

**Figure 1:**
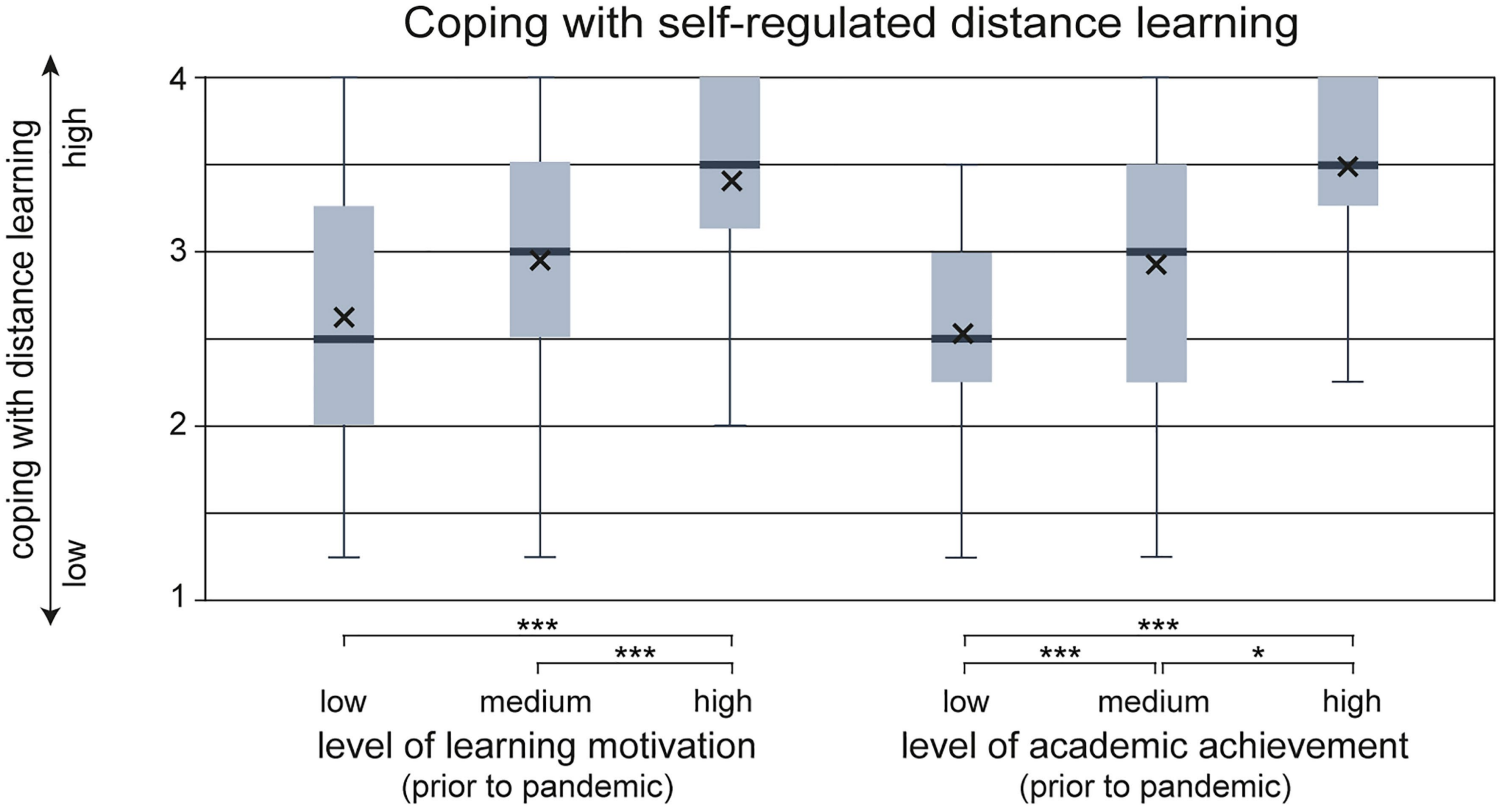
Coping with self-regulated distance learning depending on learning motivation and academic achievement prior to pandemic. Box-and-whisker plots showing the distribution of coping with self-regulated distance learning for three groups each depending on the students’ learning motivation and academic achievement prior to the pandemic. Additionally, group mean scores are shown in the form of an x. Significant differences between groups are highlighted below the diagram (^*^p<0.05 and ^***^p<0.001).

Figure 1 illustrates the distributions of coping with self-regulated distance learning depending on level of learning motivation and academic achievement, each measured prior to the COVID-19 pandemic.

Learning motivation was divided into three groups of approx. equal size (section “Learning Motivation Prior to Pandemic”). In the three groups, homogeneity of variance was asserted (*p*>0.05). The levels of coping with self-regulated distance learning differed statistically significant for the groups [*F*(2, 152)=16.574, *p*<0.001]. Tukey *post-hoc* analysis revealed a significant difference (*p*<0.001) between the mean level of coping with self-regulated distance learning in the low learning motivation group and the high learning motivation group [0.79, 95%-CI (0.46, 1.12)] as well as between the medium and the high learning motivation group [0.46, 95%-CI (0.14, 0.77)]. The low and medium learning motivation groups do not differ significantly in the mean level of coping with self-regulated distance learning. The size of the effect can be described as large (one-way ANOVA: *η*^2^=0.18).

Likewise, an analysis was conducted to assess the effects of academic achievement prior to the pandemic on self-regulated learning during the pandemic. Again, students were assigned to one of three groups according to their academic achievement (section “Academic Achievement Prior To Pandemic”). The mean level of coping with self-regulated distance learning differed statistically significant in the three groups [Welch-test *F*(2, 79.70)=27.32, *p*<0.001]. Yielding a value of *η*^2^=0.21, the effect of academic achievement was of a similar size to learning motivation. *Post-hoc* analysis using the Games-Howell test revealed a significant difference between coping with self-regulated distance learning for all groups: mean level of coping with self-regulated distance learning increased from low to medium academic achievement [0.57, 95%-CI (0.28, 0.85), *p*<0.001], from medium to high academic achievement [0.39, 95%-CI (0.05, 0.74); *p*<0.05], and from low to high academic achievement [0.96, 95%-CI (0.63, 1.29); *p*<0.001].

### Effects of Family and Child Characteristics, School Performance, and Support at Home and From School on Coping With Distance Learning

Finally, Table 3 shows the standardized β-coefficients as well as the major statistical parameters for the regression analyses, which allow for a multivariate perspective on the effects of different sets of characteristics on how well students coped with the self-regulatory demands of distance learning.

**Table 3:** Regression models explaining coping with self-regulated distance learning.

	Model 1		Model 2		Model 3	
	*β* (SE)	*t*	*β* (SE)	*t*	*β* (SE)	*t*
1. Family and child characteristics						
Parental education^a^ – level 2 (vocational training)	−0.06 (0.11)	−0.50	−0.03 (0.10)	−0.34	−0.14 (0.10)	−1.39
Parental education^a^ – level 3 (school leaving exam K12/13)	−0.10 (0.11)	−0.90	−0.11 (0.10)	−1.17	−0.15 (0.10)	−1.49
Parental education^a^ – level 4 (university degree)	−0.06 (0.10)	−0.61	0.00 (0.08)	−0.05	−0.09 (0.07)	−1.25
First language (German)	0.19 (0.07)	2.64^**^	0.14 (0.06)	2.46^*^	0.13 (0.05)	2.54^*^
Gender (female)	0.28 (0.08)	3.69^***^	0.07 (0.08)	0.93	0.04 (0.07)	0.52
2. School performance prior to lockdown						
Academic achievement			0.38 (0.07)	5.70^***^	0.22 (0.07)	3.72^**^
Learning motivation			0.26 (0.08)	3.18^**^	0.22 (0.08)	2.72^**^
3. Support at home and from school during lockdown						
Perceived support and understanding by parents					0.22 (0.07)	3.20^**^
Teacher accessibility					0.29 (0.07)	3.80^***^
*F* (df)	18.39 (5)		66.96 (7)		94.64 (9)	
*R*^2^ (adjusted)	8.2%^*^		32.0%^***^		42.3%^***^	
*N*	155		155		155	

Regression analyses conducted using MPlus, bootstrap=10,000; dependent variable coping with self-regulated distance learning in times of COVID-19.

a Reference category: level 1 – compulsory education on ISCED-level 2 only.

* *p*<0.05;

** *p*<0.01;

*** *p*<0.001.

Three models were calculated; with model 1 including solely context variables on the child and their families. The results in model 1 confirm the effect of the student’s gender, which was reported in section “Coping With Self-Regulated Distance Learning” above. As expected, girls coped more easily with self-regulated distance learning than boys. Additionally, model 1 shows a positive effect on coping with self-regulated distance learning for students with German as their first language (or one of their first languages). Due to the low numbers of students with different first languages in the region of the study (cf. section “Sample”), these findings are to be interpreted with caution. Contrary to our assumption and previous findings, this study does not show a significant effect of the highest level of parental education on self-regulated learning.

Model 2 expands this perspective by including information on school performance prior to lockdown. In line with our expectations and the findings in chapter “Effects of Learning Motivation and Academic Achievement on Coping With Distance Learning,” both variables additionally included in model 2 had a significant effect on coping with self-regulated distance learning. The increase in *R*^2^ from model 1 to model 2 by approximately 24 percentage points indicated a large effect of students’ school-related performance. Including the information on achievement and learning motivation (as continuous variables) resulted in flattening the effect of gender, implying that the girls’ increased capacity to cope with self-regulated distance learning was predominantly due to their higher academic achievement and/or learning motivation prior to lockdown.

In model 3, indicators of support at home and from school during the distance-learning period were included in the analysis. In accordance with our hypotheses, both indicators of support – accessibility of the teachers to the students during lockdown and a supportive and understanding parental behavior at home – show significant effects on coping with self-regulated distance learning. They led to an increase in *R*^2^ by another 10 percentage points, so that the third (full) model explains 42% of the variance in coping with self-regulated distance learning.

## Discussion

### Major Findings

Teachers, students, and parents suddenly faced fundamental challenges as schools were forced to switch to distance learning in spring 2020 due to the COVID-19 pandemic. Despite having almost no lead-time, our findings suggest that those challenges were met surprisingly well. The vast majority of students in our study reported that they could get in touch with their teachers easily (often and sometimes: 85.2%) and that they could access their learning materials easily (often and sometimes: 75.3%). Regarding self-regulatory demands, between 60 and 85.8% of the students had little or no problem in structuring their everyday learning and were mostly able to master the tasks given to them on their own. With respect to loneliness and social isolation, more than 90% of the students reported having been able to keep in touch with their friends (91.6%) and more than 80% felt lonely only seldom or never (82.6%). These findings also corresponded with the results of an Austria-wide study by Schober et al. (2020). At the same time, more than half of students were bored (often or sometimes: 54.2%), which can be viewed within the context of extremely restricted leisure opportunities due to lockdown, as well as in regard of the findings by Grewenig et al. (2020), Huber and Helm (2020), and Wößmann et al. (2021) pointing to very low daily study time during home schooling for some students. Summing up, students generally coped well with the demands they had to face during the first lockdown, but at the same time, it is evident how demanding the situation was, at least for some young people.

The analyses in this paper point to various groups of students who had comparatively more problems in coping with the demands of distance learning and the circumstances of lockdown and isolation. The most important factors to explain how well students were able to cope with self-regulatory demands during distance learning were their academic achievement and learning motivation prior to lockdown. This can be shown as bivariate relationships between academic achievement and learning motivation and coping with self-regulated distance learning as well as in the multivariate regression model, where school performance prior to lockdown explains by far the largest proportion of variance. Since a high level of school performance is strongly associated with competencies in self-organization (Zimmerman and Schunk, 2011; Jacob and Parkinson, 2015; Edossa et al., 2017), we assume that students with high academic achievement and learning motivation prior to the lockdown could rely on their pre-existing self-organizational skills, desires, and willingness to learn and therefore were better able to cope with the specific self-regulatory demands during distance learning.

Furthermore, this paper shows the significance of support for young people during the lockdown. This concerns firstly teachers not only as important reference points in school-related matters to support students with structuring their learning tasks but also as emotional support. Secondly, parents play an important role, as can be seen in the construct included in the regression model measuring the level of responsiveness, understanding, and willingness to provide support by parents. The present study therefore confirms the relevance of support and relationships for self-regulation (Bembenutty, 2013; Perry et al., 2018) within the context of distance learning due to a pandemic.

Overall, students with more favorable starting conditions with respect to school performance and support at home and from school report, on average, higher levels of coping with the self-regulatory demands of home schooling. Assuming that this also points to higher learning gains during distance learning, prolonged periods of having to learn at home seem to pose a severe risk of further widening educational inequalities.

### Strengths and Limitations of the Study

Our analyses are based upon a longitudinal study. Since data collection had already commenced in spring 2019, we can draw on measures for academic achievement and learning motivation collected well in advance of the pandemic. This allows the analysis of the effects of school performance prior to the lockdown on coping with the demands of distance learning.

A systematic review of 97 online studies conducted between March 2020 and November 2020 regarding distance learning during the COVID-19 pandemic (Helm et al., 2021) showed that most of the previous studies used only descriptive analyses. Therefore, this research also makes an important contribution to closing the methodological gap by using multivariate regression analyses and a longitudinal design to explain differences in coping with self-regulated distance learning.

Some limitations that relate to the regional nature of the study and the measures included in the analyses do need to be mentioned. While the sample underlying this paper is representative for the region examined, as well as for other rural-alpine regions, one must exercise caution when attempting to generalize the findings further. Most noteworthy are the specifics of population composition, resulting in a lower variance with regard to socioeconomic background (parental education) and only a small subsample of students who do not speak German as their first language. Nonetheless, the general picture, which the data show about the students’ situation during distance learning and lockdown and the factors that influence coping with the self-regulatory demands of distance learning are very similar to findings in other studies with a wider target population.

Furthermore, the study does only include a measure of coping with self-regulatory demands of distance learning but not a measure of self-regulation itself. There are also no measures of self-regulation prior to the lockdown and academic achievement during home-schooling available that would allow to answer with validity the questions if home learning was disproportionately more difficult than classroom-based learning, or academic progress was disproportionately decelerated by home learning in the low-achieving/low-motivated group of students.

## Conclusion

This paper has described students’ perceptions of the conditions under which they learned during the first strict lockdown due to the COVID-19 pandemic, which led to a complete shift to distance learning in Austrian schools from March 16, 2020. In line with research on distance learning conducted in the past months, self-regulatory demands were deemed to be one of the most important challenges for students when learning at home (e.g., APA, 2020; Grewenig et al., 2020; Huber and Helm, 2020). Hence, the paper focused upon how well students coped with self-regulatory demands during lockdown and distance learning and examined the impact of family and child characteristics, school performance as well as parental and teacher support. The findings support the hypotheses that out-of-school learning was especially challenging for students with low school performance prior to the lockdown and a lack of support at home and from school. Furthermore, they show that girls, as well as students speaking German as their first language, did cope more easily with the demands of self-regulated distance learning than boys and students with less linguistic proficiency in the national language.

In conclusion, results suggest that distance learning due to the COVID-19 pandemic has posed a substantial risk of enhancing any existing educational inequalities between low and high achieving and low- and high-motivated students. This should be taken into account when planning any further distance learning periods or when considering measures to compensate for the effects of prolonged periods of distance learning during the COVID-19 pandemic.

Although the importance of self-regulated learning becomes particularly evident in the context of distance learning, self-organizational skills are not only important in times of crisis. It can therefore be concluded that schools should focus on promoting self-regulated learning in the future.

Future research needs to be conducted in order to examine mid- and long-term effects of distance learning. The study, upon which this paper is based, aims to contribute to closing this research gap with the next survey waves of the longitudinal study to be conducted in the summer terms 2021 and 2022. A joint analysis of the data from all four survey rounds (2019–2022) will permit the examination of mid-term effects of students’ coping with the demands of distance learning on their educational attainment and psycho-social well-being.

## Data Availability Statement

The raw data supporting the conclusions of this article will be made available by the authors, without undue reservation.

## Ethics Statement

The studies involving human participants were reviewed and approved by the Department of Education, Government of the Province of Tyrol. Written informed consent to participate in this study was provided by the participants’ legal guardian/next of kin.

## Author Contributions

All authors listed have made a substantial, direct and intellectual contribution to the work, and approved it for publication.

## Conflict of Interest

The authors declare that the research was conducted in the absence of any commercial or financial relationships that could be construed as a potential conflict of interest.

## Publisher’s Note

All claims expressed in this article are solely those of the authors and do not necessarily represent those of their affiliated organizations, or those of the publisher, the editors and the reviewers. Any product that may be evaluated in this article, or claim that may be made by its manufacturer, is not guaranteed or endorsed by the publisher.

## References

[ref1] ADAS and LIFE (2020). Ergebnisse der ADAS-Corona Umfrage. ADAS-Rundbrief vom. Available at: https://adas-berlin.de/wp-content/uploads/2020/06/Ergebnisse-Umfrage-ADAS-LIFE-e.V.pdf (Accessed June 25, 2020).

[ref2] Anger S. Bernhard S. Dietrich H. Lerche A. Patzina A. Sandner M.. (2020). Schulschließungen wegen corona: Regelmäßiger Kontakt zur Schule kann die schulischen Aktivitäten der Jugendlichen erhöhen. *IAB-Forum*, April, 1–11.

[ref3] APA (2020). Psychology’s understanding of the challenges related to the COVID-19 global pandemic in the United States. *APA*.

[ref4] Baier D. Kamenowski M. (2020). Wie erlebten Jugendliche den Corona-Lockdown? Ergebnisse einer Befragung im Kanton Zürich. Züricher Fachhochschule für Angewandte Wissenschaften. Available at: https://digitalcollection.zhaw.ch/bitstream/11475/20095/3/2020_Baier-Kamenowski_Jugendliche-Corona-Lockdown.pdf (Accessed April 20, 2021).

[ref5] Bembenutty H. (2013). “The triumph of homework completion through a learning academy of self-regulation” in Application of Self-Regulated Learning across Diverse Disciplines. eds. Bembenutty H. Cleary T. J. Kitsantas A. (New York: Information Age Publishing, Inc.), 153–196.

[ref6] Berger F. Fend H. (2005). Kontinuität und Wandel in der affektiven Beziehung zwischen Eltern und Kindern vom Jugend- bis ins Erwachsenenalter. [Continuity and change in the affective relationship between parents and children from adolescence to adulthood]. Z. Soziol. Erzieh. Sozi. 25, 8–21.

[ref7] Berghammer C. (2020). Wie gut gelingt Homeschooling in der Corona-Krise? *Corona-Blog* (blog). Available at: https://viecer.univie.ac.at/corona-blog/corona-blog-beitraege/blog47/ (Accessed April 20, 2021).

[ref8] BMBWF (2020). Umgang des Bildungssystems mit dem Coronavirus – Eckpunkte [Dealing with the Corona Virus in the Education System].

[ref9] Cleary T. J. Zimmerman B. J. (2006). Teachers’ perceived usefulness of strategy microanalytic assessment information. Psychol. Sch. 43, 149–155. 10.1002/pits.20141

[ref10] Edossa A. K. Schroeders U. Weinert S. Artelt C. (2017). The development of emotional and behavioral self-regulation and their effects on academic achievement in childhood. Int. J. Behav. Dev. 42, 192–202. 10.1177/0165025416687412

[ref11] Efklides A. (2011). Interactions of metacognition with motivation and affect in self-regulated learning: the MASRL model. Educ. Psychol. 46, 6–25. 10.1080/00461520.2011.538645

[ref12] Eisenberg N. Chang L. Ma Y. Huang X. (2009). Relations of parenting style to Chinese children’s effortful control, ego resilience, and maladjustment. Dev. Psychopathol. 21, 455–477. 10.1017/S095457940900025X, PMID: 19338693 PMC2771550

[ref13] Fend H. Prester H.-G. (1986). Dokumentation der Skalen des Projekts “Entwicklung im Jugendalter.” Bericht aus dem Projekt “Entwicklung im Jugendalter”. Konstanz: Universität Konstanz; Sozialwissenschaftliche Fakultät.

[ref14] Grewenig E. Lergetporer P. Werner K. Woessmann L. Zierow L. (2020). COVID-19 and educational inequality: how school closures affect low- and high-achieving students. Collaborative Research Center Transregio Rationality & Competition. [Preprint] No. 260, 31.

[ref15] Heller S. Zügel O. (2020). “Schule zu Hause” in Deutschland. Bestandsaufnahme im Corona-Lockdown aus Perspektive der Schüler/-innen und Eltern. Zurich: Deutsche Telekom Stiftung.

[ref16] Helm C. Huber S. G. Loisinger T. (2021). Was wissen wir über schulische Lehr-Lern-Prozesse im Distanzunterricht während der Corona-Pandemie? – evidenz aus Deutschland, Österreich und der Schweiz. Z. Erziehwiss. 24, 237–311. 10.1007/s11618-021-01000-z, PMID: 33686343 PMC7931167

[ref17] Holtgrewe U. Lindorfer M. Siller C. Vana I. (2020). Von Risikogruppen zu Gestaltungschancen: Lernen im Ausnahmezustand. Momentum-Kongress (online). Available at: https://www.zsi.at/object/publication/5699/attach/LiA-Momentum20-final[1_.pdf (Accessed April 20, 2021).

[ref18] Huber S. G. Günther P. S. Schneider N. Helm C. Schwander M. Schneider J.. (2020). COVID-19 und aktuelle Herausforderungen in Schule und Bildung. Münster: Waxmann 10.31244/9783830942160

[ref19] Huber S. G. Helm C. (2020). COVID-19 and schooling: evaluation, assessment and accountability in times of crises – reacting quickly to explore key issues for policy, practice and research with the school barometer. Educ. Assess. Eval. Account. 32, 237–270. 10.1007/s11092-020-09322-y, PMID: 32837626 PMC7286213

[ref20] Illeris K. (2009). Contemporary Theories of Learning. London, New York: Routledge.

[ref21] IQS (2019). Data provided on request by Institut des Bundes für Qualitätssicherung im österreichischen Schulwesen [Federal Institute for quality assurance in the Austrian school system]. Available at: https://www.iqs.gv.at/fdb.

[ref22] Jacob R. Parkinson J. (2015). The potential for school-based interventions that target executive function to improve academic achievement. Rev. Educ. Res. 85, 512–552. 10.3102/0034654314561338

[ref23] Jesacher-Rößler L. Klein E. D. (2020). COVID-19: Strategien der Schulentwicklung in der Krise: Ergebnisse einer Schulleitungsbefragung in Österreich. Innsbruck: Universität Innsbruck. Available at: https://diglib.uibk.ac.at/5350023 (Accessed April 13, 2021).

[ref24] Korlat S. Kollmayer M. Holzer J. Lüftenegger M. Pelikan E. R. Schober B.. (2021). Gender differences in digital learning during COVID-19: competence beliefs, intrinsic value, learning engagement, and perceived teacher support. Front. Psychol. 12:637776. 10.3389/fpsyg.2021.637776, PMID: 33868109 PMC8043960

[ref25] Letzel V. Pozas M. Schneider C. (2020). Energetic students, stressed parents, and nervous teachers: a comprehensive exploration of inclusive homeschooling during the COVID-19 crisis. Open Educ. Stud. 2, 159–170. 10.1515/edu-2020-0122

[ref26] Maaz K. Baumert J. Trautwein U. (2010). “Genese sozialer Ungleichheit im institutionellen Kontext der Schule: Wo entsteht und vergrößert sich die soziale Ungleichheit?” in Bildungsungleichheit revisited: Bildung und soziale Ungleichheit vom Kindergarten bis zur Hochschule. Studien zur Schul- und Bildungsforschung 30. eds. Krüger H.-H. Rabe-Kleberg U. Kramer R.-T. Budde J. (Wiesbaden: VS, Verl. für Sozialwiss), 69–102.

[ref27] Muthen L. Muthen B. (2017). Mplus Version 8 User’s Guide. Los Angeles, CA: Muthen & Muthen.

[ref28] Neuweg G. H. (2014). Schulische Leistungsbeurteilung [Assessment and Grading in Schools]. Linz: Trauner.

[ref29] OECD (2020). How Prepared Are Teachers and Schools to Face the Changes to Learning Caused by the Coronavirus Pandemic? Teaching in Focus 2020/32. Paris: OECD Publishing.

[ref30] Pandero E. (2017). A review of self-regulated learning: six models and four directions for research. Front. Psychol. 8:422. 10.3389/fpsyg.2017.00422, PMID: 28503157 PMC5408091

[ref31] Pelikan E. R. Lüftenegger M. Holzer J. Korlat S. Spiel C. Schober B. (2021). Learning during COVID-19: the role of self-regulated learning, motivation, and procrastination for perceived competence. Z. Erzieh. 10.1007/s11618-021-01002-x, PMID: 33686344 PMC7931168

[ref32] Perry N. E. (2013). Classroom process that support self-regulation in young children. *British Journal of Educational Psychology, Monograph Series II: Psychological Aspects of Education – Current Trends*, no. 10: 45–68.

[ref33] Perry N. E. Hutchinson L. R. Yee N. Määttä E. (2018). “Advances in understanding young children’s self-regulation of learning” in Handbook of Self-Regulation of Learning and Performance. eds. Schunk D. H. Greene J. A. (New York and London: Routledge), 457–472.

[ref34] Refle J.-E. Voorpostel M. Lebert F. Kuhn U. Klaas H. S. Ryser V.-A.. (2020). First results of the Swiss household panel—Covid-19 study*. FORS Working Paper Series* paper 2020-1: 1–51.

[ref35] Rimm-Kaufman S. E. Curby T. W. Grimm K. J. Nathanson L. Brock L. L. (2009). The contribution of children’s self-regulation and classroom quality to children’s adaptive behaviors in the kindergarten classroom. Dev. Psychol. 45, 958–972. 10.1037/a0015861, PMID: 19586173

[ref36] RIS (2020). Gesamte Rechtsvorschrift für Leistungsbeurteilungsverordnung. Available at: https://www.ris.bka.gv.at/GeltendeFassung.wxe?Abfrage=Bundesnormen&Gesetzesnummer=10009375&FassungVom=2020-02-01 (Accessed August 13, 2021).

[ref37] Schober B. Lüftenegger M. Spiel C. Holzer J. Korlat Ikanovic S. Pelikan E.. (2020). Lernen unter COVID-19-Bedingungen. Erste Ergebnisse Schüler*innen. Available at: https://lernencovid19.univie.ac.at/fileadmin/user_upload/p_lernencovid19/Zwischenergebnisse_Schueler_innen.pdf (Accessed April 20, 2021).

[ref38] Schunk D. H. Greene J. A. (2018). “Historical, contemporary, and future perspectives on self-regulated learning and performance” in Handbook of Self-Regulation of Learning and Performance. Educational Psychology Handbook Series. 2nd ed (New York, NY: Routledge, Taylor & Francis Group), 1–15.

[ref39] Schunk D. H. Zimmerman B. J. (1997). Social origins of self-regulatory competence. Educ. Psychol. 32, 195–208. 10.1207/s15326985ep3204_1

[ref40] Schunk D. H. Zimmerman B. J. (eds.) (2008). Motivation and Self-Regulated Learning: Theory, Research, and Applications. New York: Taylor & Francis.

[ref41] Schwerzmann M. Frenzel S. (2020). “Umfrage zum Fernunterricht. Ergebnisse der Befragung im Juni 2020.” Presented at Medienkonferenz, Luzern, October 15. Available at: https://www.lu.ch/-/media/Kanton/Dokumente/BKD/Aktuelles/BKD_Fernunterricht_Praesentation_Ergebnisse_an_MK_2020_10_14.pdf?la=de-CH (Accessed April 20, 2021).

[ref42] SRCD (2020). Addressing inequalities in education during the COVID-19 pandemic. Statement of the evidence.

[ref43] Statistics Austria (2021). STATatlas. Source. Available at: https://www.statistik.at/atlas/?mapid=them_bildung_bildungsstand&layerid=layer1&sublayerid=sublayer0&languageid=0&bbox=972890,5738748,2027109,6361251,8 (Accessed August 13, 2021).

[ref44] Steiner M. Köpping M. Leitner A. Pessl G. (2020). COVID-19 LehrerInnenbefragung – Zwischenergebnisse. Was tun, damit aus der Gesundheitskrise nicht auch eine Bildungskrise wird? Available at: https://www.ihs.ac.at/index.php?id=1176 (Accessed April 20, 2021).

[ref45] Thies L. Klein Y. (2020). Unter Druck. Die Situation von Eltern und ihren schulpflichtigen Kindern während der Schulschließungen. Düsseldorf: Vodafone-Stiftung. Available at: https://www.vodafone-stiftung.de/wp-content/uploads/2020/04/Vodafone-Stiftung-Deutschland_Studie_Unter_Druck.pdf (Accessed April 20, 2021).

[ref46] Trültzsch-Wijnen C. Trültzsch-Wijnen S. (2020). Remote Schooling during the Covid-19 Lockdown in Austria (Spring 2020). KiDiCoTi National Report. University of Salzburg. Available at: https://eplus.uni-salzburg.at/obvusboa/content/titleinfo/5566858 (Accessed April 20, 2021).

[ref47] UNESCO (2021). Education: from disruption to recovery. UNESCO COVID-19 education response. Available at: https://en.unesco.org/covid19/educationresponse#schoolclosures

[ref48] Usher E. Schunk D. H. (2018). “Social cognitive theoretical perspective on self-regulation” in Handbook of Self-Regulation of Learning and Performance. Educational Psychology Handbook Series. eds. Schunk D. H. Greene J. A. 2nd ed (New York, NY: Routledge, Taylor & Francis Group), 19–35.

[ref49] Vuorikari R. Velicu A. Chaudron S. Cachia R. Di Gioia R. (2020). How Families Handled Emergency Remote Schooling During the Time of Covid Lockdown in spring 2020: Summary of Key Findings From Families With Children in 11 European Countries. Luxemburg: Publications Office of the European Union. Available at: https://data.europa.eu/doi/10.2760/31977 (Accessed April 13, 2021).

[ref50] Wacker A. Unger V. Rey T. (2020). “Sind doch Corona-Ferien, oder nicht?” in Langsam vermisse ich die Schule …. eds. Fickermann D. Edelstein B. (Münster: Waxmann), 79–94.

[ref51] Wildemann A. Hosenfeld I. (2020). Bundesweite Elternbefragung zu Homeschooling während der Covid 19-Pandemie. Erkenntnisse zur Umsetzung des Homeschoolings in Deutschland. Available at: http://www.zepf.eu/wp-content/uploads/2020/06/Bericht_HOMEschooling2020.pdf (Accessed April 19, 2021).

[ref52] Wirth J. Leutner D. (2008). Self-regulated learning as a competence: implications of theoretical models for assessment methods. Z. Psychol. 216, 102–110. 10.1027/0044-3409.216.2.102

[ref53] World Health Organization (2020). Coronavirus disease (COVID-2019) situation reports. Available at: https://www.who.int/docs/default-source/coronaviruse/situation-reports/20200311-sitrep-51-covid-19.pdf?sfvrsn=1ba62e57_10 (Accessed August 13, 2021).

[ref54] Wößmann L. Freundl V. Grewenig E. Lergetporer P. Werner K. Zierow L. (2021). Bildung in der Coronakrise: Wie haben die Schulkinder die Zeit der Schulschließungen verbracht, und welche Bildungsmaßnahmen befürworten die Deutschen? ifo Schnelldienst 73, 25–39.

[ref55] Zimmerman B. J. (2008). Investigating self-regulation and motivation: historical background, methodological developments, and future prospects. Am. Educ. Res. J. 45, 166–183. 10.3102/0002831207312909

[ref56] Zimmerman B. J. Schunk D. H. (Eds). (2011). “Self-regulated learning and performance” in Handbook of Self-Regulation of Learning and Performance. Educational Psychology Handbook Series. eds. Zimmerman B. J. Schunk D. H. (New York, NY: Routledge, Taylor & Francis Group), 1–12.

